# Feasibility and Clinical Outcomes of a 6-Month Home-Based Telerehabilitation Program in Patients with Peripheral Arterial Disease: A Prospective Single-Arm Study

**DOI:** 10.3390/life16101611

**Published:** 2026-09-25

**Authors:** Andreea Rotundu, Mihai Roca, Alexandra Maștaleru, Andra Oancea, Gabriela Popescu, Alexandru-Dan Costache, Costin Chirica, Theodor Constantin Stamate, Cristina Grosu, Florin Mitu, Maria Magdalena Leon

**Affiliations:** 1 Grigore T. Popa University of Medicine and Pharmacy Iasi, 700115 Iasi, Romania; rotundu.d.andreea@umfiasi.ro (A.R.); alexandra.mastaleru@umfiasi.ro (A.M.); andra.oancea@umfiasi.ro (A.O.); gabriela.popescu@umfiasi.ro (G.P.); dan-alexandru.costache@umfiasi.ro (A.-D.C.); chirica.costin@d.umfiasi.ro (C.C.); stamate.theodor@gmail.com (T.C.S.); mitu.florin@yahoo.com (F.M.); maria.leon@umfiasi.ro (M.M.L.); 2 Clinical Rehabilitation Hospital, 700661 Iasi, Romania; fcristina_ro@yahoo.com; 3 Department of Neurology, Grigore T. Popa University of Medicine and Pharmacy, 700115 Iași, Romania; 4 Romanian Academy of Medical Sciences, 030167 Bucharest, Romania; 5 Academy of Romanian Scientists, Ilfov 3, 050044 Bucharest, Romania

**Keywords:** peripheral arterial disease, telerehabilitation, home walking program, digital health, exercise adherence, wearable tracking

## Abstract

(1) Background: Peripheral arterial disease (PAD) is associated with substantial cardiovascular morbidity and functional impairment. This study evaluated the feasibility and adherence of a 6-month home-based telerehabilitation (TR) program and longitudinal changes in functional capacity, health-related quality of life (HRQoL), and selected cardiometabolic parameters. (2) Methods: This prospective, single-arm study enrolled 42 patients with PAD. Daily ambulation was monitored using a Xiaomi Smart Band 8, and participants received weekly telephone counseling. Assessments were performed at baseline, 3 months, and 6 months. (3) Results: Median daily step count increased from 5868 to 6905 steps/day (*p* < 0.001), whereas attainment of progressively increasing weekly targets decreased from 58.33% to 16.66% (*p* < 0.001). Median 6 min walk test (6MWT) distance increased from 390 m to 445 m at 6 months, a 55 m increase (*p* = 0.001). HRQoL, systolic blood pressure, body mass index, and total cholesterol showed significant longitudinal changes, while resting ankle–brachial index and selected cardiopulmonary exercise testing parameters remained relatively stable (*p* > 0.05). (4) Conclusions: The TR program was feasible and accompanied by increased activity and improved walking capacity. The divergence between absolute activity and target attainment supports multidimensional adherence assessment. Causal effects cannot be established given the single-arm design.

## 1. Introduction

### 1.1. Peripheral Arterial Disease: Clinical Burden and the Role of Exercise Therapy

Peripheral arterial disease (PAD) of the lower limbs is a common manifestation of systemic atherosclerosis (ATS), characterized by arterial stenosis or occlusion that reduces blood flow to the lower-extremity muscles. PAD represents an important global health problem because of its substantial burden of cardiovascular events, functional impairment, and disability. Recent estimates based on the Global Burden of Disease Study indicate that approximately 114 million people were living with PAD worldwide in 2021, while the number of prevalent cases has increased substantially over recent decades, largely reflecting population ageing and the increasing prevalence of cardiovascular risk factors. PAD is also associated with a considerable burden of disability and mortality, emphasizing the importance of strategies aimed not only at reducing cardiovascular risk but also at preserving functional capacity and quality of life [1].

In symptomatic patients, impaired lower-extremity perfusion may lead to intermittent claudication (IC), while chronic ischemia, endothelial dysfunction, inflammation, oxidative stress, and skeletal muscle abnormalities further contribute to impaired exercise tolerance [2]. During walking, these abnormalities result in an imbalance between the metabolic demands of skeletal muscle and oxygen delivery, leading to recurrent ischemia, muscle fatigue, and pain. Repeated exercise can, however, induce peripheral and muscular adaptations that improve exercise tolerance. Proposed mechanisms include improvements in endothelial function, microvascular adaptations, skeletal muscle metabolism, and oxygen utilization, which may contribute to delayed symptom onset and improved walking performance [2,3,4].

Exercise therapy is therefore a cornerstone of contemporary PAD management. The 2024 European Society of Cardiology (ESC) Guidelines for the management of peripheral arterial and aortic diseases recommend structured exercise as an integral component of the treatment of symptomatic PAD. Supervised exercise therapy (SET) has been consistently associated with improvements in walking performance, functional capacity, and health-related quality of life (HRQoL). However, despite its established clinical benefits, SET remains underused in routine practice. The 2024 ESC Guidelines therefore indicate that home-based exercise training (HBET) should be considered when SET is unavailable, while emphasizing the importance of monitoring and individualized exercise prescription [5].

### 1.2. Adherence and Access to Exercise-Based Rehabilitation in Peripheral Artery Disease

Although exercise-based rehabilitation is recommended for patients with PAD, maintaining long-term participation remains challenging. Adherence to SET may be affected by travel requirements, financial costs, geographical accessibility, availability of specialized rehabilitation facilities, and the time required to attend repeated supervised sessions [6,7]. These barriers are particularly relevant for patients who live far from specialized centers or have functional, socioeconomic, or logistical limitations.

HBET has consequently emerged as an alternative approach for increasing access to exercise rehabilitation. Evidence from randomized trials and recent meta-analytic research indicates that structured home-based walking programs can improve functional walking capacity in patients with PAD [8,9,10]. An individual participant data meta-analysis demonstrated that home-based walking exercise was associated with a clinically relevant improvement in 6 min walk distance and may provide functional benefits comparable to, or in some outcomes greater than, those achieved with supervised treadmill exercise [9]. Current international guidelines consequently support HBET when SET is not available, while emphasizing that exercise should be individualized and that monitoring and behavioral support may improve its effectiveness and adherence [5].

Technology-supported HBET may further facilitate patient self-monitoring and communication with healthcare professionals. Wearable activity monitors can provide objective information regarding daily physical activity, while telephone counseling can provide feedback, reinforce behavioral goals, and facilitate progressive adjustment of exercise targets [6,7]. However, technology alone does not necessarily ensure adherence or clinically meaningful improvement. The effectiveness of technology-supported interventions may depend on the intensity of professional support, the characteristics of the patient population, the monitoring strategy, and the reproducibility of the exercise prescription [6,7,11].

### 1.3. Telerehabilitation as a Potential Model for Improving Access to Exercise Therapy

Telerehabilitation (TR) refers to the remote delivery of rehabilitation assessment, exercise prescription, monitoring, education, and behavioral support through information and communication technologies. In cardiovascular rehabilitation, TR has increasingly been investigated as a means of extending rehabilitation beyond specialized centers, particularly for patients who experience barriers to conventional center-based programs [11,12,13,14]. Therefore, the present study aimed to assess the feasibility and adherence associated with a 6-month home-based telerehabilitation program incorporating wearable activity monitoring and structured telephone counseling in patients with PAD. In addition, we sought to characterize longitudinal changes in walking capacity, IC onset, HRQoL, and selected cardiovascular and metabolic parameters over the 6-month intervention period.

In PAD, the potential value of TR is particularly relevant because exercise therapy requires sustained participation over time, whereas access to supervised programs remains limited. Recent studies of monitored home-based exercise programs using digital technologies, behavioral support, and telephone counseling have demonstrated improvements in walking performance and quality of life, while also showing that remote monitoring can support long-term participation [6,7]. More recent telehealth-based research has also highlighted the feasibility of remote exercise and behavioral interventions in PAD populations with limited access to conventional rehabilitation services [15,16].

Nevertheless, important gaps remain in the evidence. First, existing studies have used heterogeneous home-based and technology-supported interventions, differing in exercise prescription, monitoring methods, behavioral support, and definitions of adherence. Second, the available evidence is stronger for home-based exercise generally than for low-resource TR models combining wearable activity monitoring with structured telephone counseling. Third, many studies have focused primarily on functional outcomes, while less is known about the feasibility of integrating objective daily activity monitoring, individualized progression of walking activity, and repeated clinical assessment over a longer period in routine cardiovascular rehabilitation settings. In addition, adherence is not consistently defined across studies, making comparisons between different TR approaches difficult.

The hypothesis was that participation in the intervention would be associated with improvements in walking performance and cardiovascular outcomes, along with greater adherence to this type of cardiovascular rehabilitation.

## 2. Materials and Methods

### 2.1. Study Design and Setting

This was a prospective, longitudinal, single-arm, single-center study conducted between January 2023 and January 2025 in a cardiovascular rehabilitation clinic. During the study period, 7411 patients were admitted to the clinic, of whom 179 were identified as having PAD based on the corresponding diagnostic code. Following application of the predefined eligibility criteria, 42 patients with PAD were enrolled and followed for 6 months within a home-based TR program (Figure 1).

The study was retrospectively registered on ClinicalTrials.gov (NCT07825168).

The study was conducted in accordance with the principles of the Declaration of Helsinki and was approved by the Ethics Committee under approval number 25/16.11.2022. All participants signed an informed consent form prior to inclusion.

### 2.2. Participants and Eligibility Criteria

Participants were eligible for inclusion if they met the following criteria:

Inclusion criteria:

1. Age ≥ 18 years;
2. Diagnosis of PAD, defined by an ankle–brachial index (ABI) ≤ 0.90, arterial stenosis > 70% documented by angiography, or previous lower-extremity revascularization;
3. Ability to safely perform walking exercise at home;
4. Ability to use the activity-monitoring device and read the number of daily steps displayed on the device;
5. Access to and ability to use a mobile phone for telephone counseling;
6. Ability to complete the activity journal;
7. Provision of written informed consent.

Exclusion criteria:

1. Chronic limb-threatening ischemia (CLTI);
2. Locomotor disability preventing participation in the walking exercise program;
3. Significant visual or hearing impairment interfering with participation in the intervention;
4. Moderate-to-severe cognitive impairment, defined as a Mini-Mental State Examination (MMSE) score < 17 [17];
5. Requirement for long-term oxygen therapy;
6. Incomplete medical records;
7. Inability to provide reliable information required for study assessments (Figure 1). PAD clinical presentation was classified according to the 2024 ESC Guidelines for the management of peripheral arterial and aortic diseases as symptomatic PAD related to exertion or asymptomatic PAD [5]. In patients with symptomatic PAD, the presence of IC was recorded based on clinical assessment, including its laterality (right lower limb, left lower limb, or bilateral involvement). Patients with unilateral or bilateral IC were eligible for inclusion.

### 2.3. Study Outcomes

The primary objective of this study was to evaluate the feasibility, adherence, and implementation of a 6-month home-based TR program in patients with PAD.

The secondary objectives were to analyse the long-term trends in functional capacity, the onset threshold for IC, HRQoL and selected cardiovascular and metabolic parameters over the 6-month follow-up period.

### 2.4. Baseline Clinical and Paraclinical Assessment

At enrollment, all participants underwent a standardized clinical assessment including medical history, CVR assessment, medication review, physical examination, and evaluation of PAD symptoms.

Anthropometric measurements included body mass index (BMI, kg/m^2^). Blood pressure was measured using an Omron HEM-907 automated blood pressure monitor (Omron Healthcare, Kyoto, Japan), and systolic blood pressure (SBP, mmHg) and diastolic blood pressure (DBP, mmHg) were recorded.

Laboratory investigations included fasting glucose, glycated hemoglobin (HbA1c, %), total cholesterol (TC, mg/dL), high-density lipoprotein cholesterol (HDL-C, mg/dL), low-density lipoprotein cholesterol (LDL-C, mg/dL), triglycerides (TG, mg/dL), C-reactive protein (CRP, mg/dL), white blood cell count (WBC, number/mm^3^), erythrocyte sedimentation rate (ESR, mm/h), hemoglobin (Hb, g/dL), and serum iron (µg/dL). Exploratory laboratory assessments additionally included serum creatinine (Cr, mg/dL), estimated creatinine clearance (Cl Cr, mL/min), urea (mg/dL), and uric acid (mg/dL).

The 10-year CVR (%) was estimated using the 2013 ACC/AHA Pooled Cohort Equations (ASCVD Risk Estimator [18]). The risk calculation incorporated age, sex, TC, HDL-C, SBP, antihypertensive treatment status, smoking status, and diabetes status [18].

ABI was measured bilaterally using the Fukuda Denshi VaSera VS-2000 Vascular Assessment System (Fukuda Denshi Co., Ltd., Tokyo, Japan). ABI was calculated as the ratio of ankle SBP to brachial SBP for each lower limb. Carotid ultrasound was performed using a Toshiba Aplio 500 Series ultrasound system (Toshiba Medical Systems Corporation, Ōtawara, Tochigi, Japan) to assess carotid intima-media thickness of the internal carotid arteries (IMT ICA, mm) and the presence of carotid atherosclerotic plaques.

The same clinical and paraclinical procedures were repeated at 3 and 6 months.

### 2.5. Functional Capacity and Cardiopulmonary Exercise Testing

Functional capacity was assessed at baseline, 3 months, and 6 months using cardiopulmonary exercise testing (CPET) and the 6 min walk test (6MWT). The same assessment procedures were applied at each evaluation time point.

CPET was performed using BTL CardioPoint software (version 2.32; BTL Industries, Prague, Czech Republic) and a compatible electronically braked cycle ergometer (Ergoline Ergoselect 100; ergoline GmbH, Bitz, Germany). The protocol consisted of a progressive, symptom-limited maximal exercise test. Following a baseline period, patients started at an initial workload of 10 W, with a continuous ramp increase of 15 W/min tailored to achieve an exercise duration of 8–12 min. A 10 min recovery phase was monitored post-exercise. Continuous 12-lead electrocardiography provided real-time cardiac monitoring. Blood pressure was measured at two-minute intervals via the manual auscultatory method. During the test, heart rate (HR), exercise workload expressed in watts (W), oxygen consumption (VO2) and the minute ventilation–carbon dioxide production (VE/VCO_2_) ratio were the main monitored parameters. An individualized target HR was determined during the baseline CPET, as the HR corresponding to the first anaerobic threshold, identified by the V-slope method. This value was subsequently utilized as the clinical reference for prescribing personalized exercise intensity during the home-based TR program.

The 6MWT was performed to assess functional walking capacity. The total distance walked during the test was recorded and expressed both as the absolute distance achieved, in meters, and as a percentage of the predicted distance. The predicted 6MWT distance was calculated using sex-specific reference equations. For male participants, the predicted distance was calculated as: 867 − (5.71 × age) + (1.03 × height). For female participants, the predicted distance was calculated as: 525 − (2.86 × age) + (2.71 × height) − (6.22 × BMI) [19]. Age was expressed in years, height in centimeters, and BMI in kg/m^2^. The percentage of predicted distance achieved was calculated for each participant as the ratio between the observed and predicted 6MWT distance, multiplied by 100. Patient-reported symptoms occurring during the 6MWT were recorded. HR, blood pressure, and peripheral oxygen saturation (SpO_2_) were assessed immediately before and after the test.

### 2.6. Health-Related Quality of Life Assessment

HRQoL was assessed using three PAD-specific patient-reported outcome instruments: the Peripheral Artery Disease Quality of Life Questionnaire (PADQOL), the Quality of Life questionnaire for patients with Peripheral Artery Disease (QOLPAD), and the Vascular Quality of Life Questionnaire-6 (VascuQoL-6). The questionnaires were administered at baseline, 3 months, and 6 months, with the investigator available to clarify procedural aspects without influencing participants’ responses.

The PADQOL is a validated 38-item PAD-specific questionnaire assessing the physical, psychosocial, and emotional impact of the disease. The instrument comprises five main factors: social relationships and interactions, self-concept and feelings, symptoms and limitations in physical functioning, fear and uncertainty, and positive adaptation. Three additional items address sexual function, intimate relationships, and employment. The original validation study demonstrated good internal consistency, with Cronbach’s alpha coefficients ranging from 0.73 to 0.92 across the five factors, while correlations with conceptually related measures ranged from 0.40 to 0.80, supporting convergent validity [20].

The QOLPAD is a validated 12-item disease-specific questionnaire developed specifically for patients with PAD. It assesses disease burden across aspects of physical and mental health, participation in everyday activities, working life, leisure and social activities, treatment-related burden, and psychological aspects of living with PAD. Items refer to the preceding 7 days and are rated using five-point response scales according to the specific item. The validation study demonstrated satisfactory internal consistency, with Cronbach’s alpha coefficients of 0.74 at baseline and 0.84 at follow-up. Convergent validity was supported by correlations with the VascuQoL global score of −0.77 at baseline and −0.87 at follow-up [21].

The VascuQoL-6 is a validated 6-item short-form instrument derived from the original VascuQoL and designed to assess disease-specific quality of life in patients with PAD. Its items address activity limitations, symptoms and leg-related difficulties, walking ability, emotional concerns related to impaired circulation, social participation, and pain-related discomfort. The VascuQoL-6 has demonstrated good internal consistency, with Cronbach’s alpha coefficients of 0.85 before and 0.94 after intervention. Its score correlated strongly with measured walking ability (r = 0.72, *p* < 0.001) and demonstrated good responsiveness to clinical change, with a standardized response mean of 1.15 [22].

### 2.7. Home-Based Telerehabilitation Intervention

The intervention consisted of a 6-month structured home-based walking program supported by wearable activity monitoring and weekly telephone counseling.

At enrollment, the walking program was individually prescribed according to the participant’s clinical and functional status. Participants were instructed to perform regular walking exercise and to progressively increase their daily activity.

The initial daily activity level was established from the participant’s baseline activity data. During weekly telephone consultations, the investigator reviewed the previous 7 days of activity, assessed general health status and tolerance to exercise, and provided individualized recommendations for progression.

Participants were encouraged to increase their average daily step count by approximately 250 steps relative to the previous week’s average. When this increase could not be achieved, participants were advised to maintain an upward trend relative to their previous activity level.

Exercise intensity was individualized using the target HR established during the baseline CPET. Participants were instructed to walk until reaching the individualized target HR.

When IC developed during walking, participants were instructed to continue walking after the onset of symptoms until reaching a tolerable mild-to-moderate level of claudication discomfort, characterized by noticeable leg pain that remained manageable and did not require stopping. Participants were instructed to stop or rest if the pain became severe or intolerable. Exercise was therefore not intended to continue to maximal or intolerable pain. The intervention did not involve real-time video supervision. Instead, remote monitoring consisted of wearable activity tracking and weekly telephone counseling.

### 2.8. Wearable Activity Monitoring

Physical activity was monitored using the Xiaomi Smart Band 8, a commercially available wrist-worn activity tracker. The device was selected because of its accessibility, ease of use, and suitability for long-term monitoring in a home-based rehabilitation setting. Participants received standardized instructions on device use at enrollment and were instructed to wear the device during daily activities throughout the intervention period.

Previous research has evaluated the criterion validity and accuracy of Xiaomi Smart Band devices for step counting. Alvarenga et al. reported good agreement between step counts recorded by a Xiaomi Smart Band and reference measurements in individuals after stroke, although accuracy varied according to walking conditions and gait characteristics [23]. These findings support the use of Xiaomi Smart Band devices as practical tools for monitoring changes in daily physical activity, while recognizing that step counts may be influenced by individual characteristics, walking speed, and movement patterns.

Participants were instructed to review their daily step counts and record their activity data in an activity journal. The recorded step count reflected total daily ambulation rather than exclusively prescribed walking exercise and could therefore include walking performed at home, during routine daily activities, and outside the home.

Because the Xiaomi Smart Band 8 is a wrist-worn activity monitor, step counts may be affected by non-ambulatory arm movements as well as by slow walking or other walking conditions. Therefore, recorded step counts were used primarily to monitor longitudinal changes in overall physical activity and adherence to the prescribed program rather than to quantify the exact dose of exercise.

### 2.9. Adherence and Feasibility Outcomes

Adherence was assessed using several activity-based indicators derived from the weekly step records. The indicators included:

1. Percentage of weeks in which the participant achieved the average daily step target established for the following week;
2. Percentage of weeks in which the participant increased average daily steps relative to the previous week;
3. Percentage of weeks in which the participant exceeded an average of over 5000 steps a day;
4. Percentage of weeks in which the participant exceeded an average of over 10,000 steps a day;
5. Median average daily step count during each 12-week period.

This multidimensional approach was chosen because a progressively increasing exercise prescription may make the achievement of a predefined weekly target more difficult over time even when the participant’s absolute physical activity continues to increase.

Feasibility was additionally considered in relation to the ability to implement the intervention over 6 months, completion of scheduled monitoring contacts, and retention in the intervention.

### 2.10. Statistical Analysis

Continuous variables will be presented as medians and interquartile ranges (IQR) due to their non-normal distribution, while categorical variables will be expressed as absolute frequencies and percentages (*n*, %).

Statistical analyses were performed using IBM SPSS Statistics (version 26.0; IBM Corp., Armonk, NY, USA). Non-normal distribution of continuous data was confirmed via Shapiro–Wilk testing and visual inspection. Longitudinal changes across the three assessment periods were analyzed using the Friedman test, with test results reported via the Chi-square (χ^2^) statistic. Significant overall effects (*p* < 0.05) were followed by post–hoc pairwise comparisons using the Wilcoxon signed-rank test. To quantify these pairwise changes, median differences and 95% confidence intervals (CI) were calculated using the Hodges–Lehmann estimator. Post hoc *p*-values were adjusted using the Bonferroni correction to control the family-wise error rate. Effect sizes for the pairwise comparisons were quantified using the Wilcoxon r effect size, calculated as r = |Z|/√*n* and interpreted according to standard thresholds.

An a priori sample size calculation was performed using G*Power (version 3.1.9.7). Based on a repeated-measures design across three time points (0, 3, and 6 months) evaluating non-parametric continuous variables, the minimum required sample size was determined to be 35 participants. To account for the use of non-parametric tests (Friedman and Wilcoxon signed-rank tests), this estimate was adjusted using Lehmann’s Asymptotic Relative Efficiency (ARE) by applying a correction factor of 1.15, resulting in a target sample size of 41 subjects. Ultimately, a convenience sample of all consecutive eligible patients during the study enrollment window was recruited, resulting in a final sample size of *n* = 42, thereby exceeding the minimum statistical power threshold.

## 3. Results

### 3.1. General Characteristics of Patients and Comorbidities

The study cohort comprised predominantly male participants (73.8%), with a mean duration since peripheral artery disease (PAD) diagnosis of less than one year in 40.4% of cases. The majority presented with symptomatic exertional PAD (88.1%), and regarding lifestyle factors, 54.7% were former smokers while 26.2% were current smokers (Table 1).

The cohort was characterized by a high burden of cardiovascular comorbidities. Hypertension, dysglycemia, dyslipidemia, and overweight or obesity affected approximately 80% or more of participants, while chronic kidney disease (CKD) and heart failure were less frequent (Figure 2).

### 3.2. Specific Treatment for Peripheral Arterial Disease

Regarding medication, at the time of enrolment in the study, 50% of participants were receiving oral treatment with Cilostazol or Pentoxifylline. The other half were prescribed oral Cilostazol or Pentoxifylline for home treatment at the first evaluation, and no major changes in PAD pharmacotherapy were observed during the study period.

At the time of enrollment in the study, most participants were already receiving optimized cardiovascular treatment: 59.5% were on antiplatelet therapy, and 92.8% were on antihypertensive medication, both of which were continued throughout the 6-month follow-up period. Regarding lipid-lowering therapy, 78.5% of patients were already taking statins, and treatment was recommended for the remaining 21.5% during the initial evaluation. In accordance with current guidelines, high-intensity statin therapy with Atorvastatin (40–80 mg/day) or Rosuvastatin (20–40 mg/day) was recommended. Furthermore, at the initial assessment, patients received counseling from a dietitian regarding specific dietary recommendations.

### 3.3. Clinical and Paraclinical Outcomes During the 6 Month Follow-Up

Statistically significant differences were observed in SBP values among the three assessment time points (Chi-square = 10.126, *p* = 0.006). Post hoc analysis showed that this difference was driven by significant reductions in SBP between the initial and 3-month assessments, and between the initial and 6-month assessments (Table 2). In contrast, the comparison between the 3-month and 6-month assessments showed no statistically significant differences, suggesting that the reduction in SBP achieved during the first 3 months was maintained through the end of the follow-up period, with no further significant changes.

Regarding BMI, statistically significant differences were identified among the three assessment time points (Chi-square = 22.817, *p* < 0.001). Post hoc analysis demonstrated significant reductions between the baseline and 3-month assessments and between the 3- and 6-month assessments, indicating a progressive decrease in BMI over the course of the intervention. Consequently, the comparison between the initial and 6-month assessments revealed the greatest reduction in BMI, confirming that the intervention’s effect was maintained and amplified over time (Table 2).

Statistically significant differences were found in TC levels among the three assessment time points (Chi-square = 6.650, *p* = 0.036). Post hoc analysis showed significant reductions in TC from baseline to the 3-month and 6-month assessments (Table 2). In contrast, no statistically significant changes were observed between the 3-month and 6-month assessments, suggesting that the reduction in total cholesterol occurred during the early phase of the intervention and was subsequently maintained until the final assessment.

No significant longitudinal changes were observed in glycemic parameters (fasting glucose and HbA1c), HDL-C, LDL-C, TG, CRP, WBC, ESR, Hb, or serum iron (*p* > 0.05 for all variables; Table 2). Similarly, no significant changes were observed in the exploratory renal parameters, including Cr, Cl Cr, urea, and uric acid (*p* > 0.05 for all variables). Furthermore, the IMT ICA did not change significantly over the follow-up period. Consequently, no post hoc comparisons were performed (Table 2).

### 3.4. Assessment of Functional Status, Health-Related Quality of Life, and Cardiovascular Risk

Regarding the IC onset threshold, significant differences were observed among the three assessment time points (Chi-square = 55.866, *p* < 0.001). Post hoc analysis showed significant increases from the initial to the 3-month, 3- to 6-month, and initial to 6-month assessments (Table 3). These results indicate a progressive improvement in the distance traveled before the onset of CI symptoms over the course of the 6-month TR program.

Similarly, for the total distance covered on the 6MWT, â significant differences were found among the three assessments (Chi-square = 14.746, *p* = 0.001). Post hoc analysis showed significant differences between the 3- and 6-month assessments, as well as between the baseline and 6-month assessments, whereas no statistically significant changes were observed between the baseline and 3-month assessments (Table 3). These results suggest that improvements in exercise capacity became evident after a longer period of intervention.

A similar pattern was observed for the percentage of the predicted distance achieved on the 6MWT, where significant differences were detected among the three assessment time points (Chi-square = 17.568, *p* < 0.001). Post hoc analysis demonstrated significant increases from the 3- to the 6-month assessments and from baseline to the 6-month assessment, with no significant differences between the baseline and 3-month assessments (Table 3), suggesting a progressive improvement in functional performance.

With regard to the estimated 10-year CVR (CVR at 10 years), significant differences were identified between the three assessment time points (Chi-square = 7.840, *p* = 0.020). Post hoc analysis showed a significant reduction in CVR at 10 years between the initial and 6-month assessments, whereas comparisons between the initial and 3-month assessments and between the 3- and 6-month assessments did not reveal any statistically significant differences (Table 3). These results suggest that the reduction in CVR became evident only after the TR program was completed.

The right and left ABI values, as well as the parameters assessed during CPET (maximum exercise intensity (Watts), VE/VCO_2_, and target heart rate (HR) did not show statistically significant changes between the three assessment time points (*p* > 0.05 for all variables; Table 3). Consequently, no post hoc comparisons were performed, as the results indicated that these parameters remained relatively stable throughout the 6-month follow-up period.

All three questionnaires used to assess HRQoL showed significant improvements over the course of the TR program. For the PADQOL, significant differences were observed among the three assessment time points (Chi-square = 33.365, *p* < 0.001), and the post hoc analysis revealed significant improvements across all assessment time points. Similarly, the QOLPAD showed significant increases both between the initial and 3-month assessments, between the 3- and 6-month assessments, and between the initial and 6-month assessments (Chi-square = 19.557, *p* < 0.001), suggesting a progressive improvement in HRQoL. For the VascuQoL-6, statistically significant differences emerged (Chi-square = 31.453, *p* < 0.001). Post hoc analysis showed significant improvements from baseline to the 3-month assessment and from the 3-month to the 6-month assessment, whereas the difference from baseline to the 6-month assessment did not reach statistical significance (Table 4).

### 3.5. Assessment of Adherence to the Telerehabilitation Program

All 42 participants remained enrolled in the 6-month TR program. Four participants did not attend the scheduled 3-month clinical reassessment, and two did not attend the scheduled 6-month clinical reassessment. These missed visits were considered missing outcome assessments and did not represent discontinuation of the TR intervention.

During the first 3 months, participants achieved the average daily step target established for the following week during 58.33% of the observed periods. During the final 3 months, this proportion decreased to 16.66% (*p* < 0.001). Similarly, the proportion of weeks in which participants increased their average daily step count relative to the previous week decreased from 66.67% to 50.00% (*p* < 0.001) (Table 5).

In contrast, indicators of absolute physical activity showed an opposite pattern. The proportion of weeks in which participants exceeded an average of 5000 steps/day increased from 75.00% during the first 3 months to 91.66% during the final 3 months (*p* = 0.048). The proportion of weeks in which participants exceeded an average of 10,000 steps/day also increased significantly (*p* = 0.007). Median average daily step count increased from 5868.00 steps/day during the first 3 months to 6905.00 steps/day during the final 3 months (*p* < 0.001) (Table 5).

Overall, adherence indicators showed a divergent pattern: achievement of progressively increasing weekly targets declined over time, whereas absolute daily physical activity increased.

## 4. Discussion

### 4.1. Principal Findings

The primary objective of this study was to assess the feasibility of a 6-month home-based TR program in patients with PAD, with adherence to the walking intervention defined as the primary feasibility outcome.

The principal finding was that the program could be implemented over a 6-month period using a relatively simple model combining progressive walking, wearable activity monitoring, and weekly telephone counseling. Overall physical activity increased during the second half of the intervention, as demonstrated by the increase in median daily step count and the greater proportion of weeks in which the participant exceeded an average of over 5000 steps/day.

At the same time, achievement of the progressively increasing weekly step targets declined. Therefore, adherence should not be interpreted as uniformly high or uniformly declining. Instead, the data suggest that participants increased their absolute physical activity while progressively demanding targets became more difficult to achieve.

Secondary exploratory analyses showed significant improvements in the IC onset threshold, 6MWT distance, and disease-specific quality of life measures. ABI and the selected CPET parameters remained relatively stable.

Because the study had no control group, these longitudinal changes cannot be interpreted as direct effects of the TR intervention.

### 4.2. Feasibility and Intervention Delivery

Adherence represents an important challenge in long-term exercise rehabilitation for patients with PAD. Conventional SET may be constrained by travel requirements, treatment-related costs, geographical barriers, and limited access to specialized rehabilitation facilities. In contrast, home-based walking interventions incorporating behavioral support may facilitate participation by allowing patients to perform exercise within their usual environment. Previous randomized evidence has demonstrated that structured home-based walking interventions can improve walking behavior compared with usual care, supporting their potential applicability in PAD [24].

The present intervention was designed to enhance feasibility by transferring most of the rehabilitation process to the patient’s home. The use of a wearable device provided objective information on daily physical activity, while weekly telephone counseling offered individualized behavioral support and enabled the exercise prescription to be progressively adjusted according to each participant’s activity level. This approach reduced the need for frequent face-to-face visits while maintaining regular contact between participants and the rehabilitation team.

The feasibility of the intervention was also supported by the ability to collect repeated physical activity data throughout the six-month follow-up. The combination of wearable-based monitoring and periodic telephone counseling provided a practical framework for remotely supervising walking activity and adjusting individualized exercise targets over time.

Adherence was assessed using several complementary indicators, including achievement of individualized weekly step targets, changes in activity relative to the preceding week, overall daily step volume, and attainment of predefined activity thresholds. The detailed results and interpretation of these adherence indicators are presented in Section 4.10.

### 4.3. Blood Pressure and Metabolic Parameters

SBP decreased significantly during follow-up, with the largest change occurring during the first 3 months. This pattern is biologically plausible in the context of regular aerobic exercise, which may influence blood pressure through several mechanisms, including reductions in sympathetic nervous system activity and improvements in vascular endothelial function. A recent systematic review and meta-analysis reported beneficial effects of aerobic and resistance exercise on sympathetic activity in patients with hypertension [25], while another meta-analysis of randomized controlled trials demonstrated improvements in vascular function following exercise in patients with hypertension [26]. Evidence from earlier meta-analytic work also supports the blood-pressure-lowering effect of exercise training [27].

The reduction in SBP observed in the present study should nevertheless be interpreted cautiously. The absence of a significant difference between 3 and 6 months suggests that most of the observed reduction occurred during the initial phase and was subsequently maintained rather than continuously amplified. Moreover, participants were receiving cardiovascular pharmacological treatment, and medication adjustments were not experimentally controlled. Therefore, the observed reduction in SBP cannot be attributed specifically to TR.

BMI also decreased progressively, with the most evident change occurring at 6 months. This finding is compatible with previous evidence indicating that sustained exercise can contribute to reductions in body weight, particularly when the intervention is maintained over several months [28]. The clinical relevance of this finding is potentially greater in PAD because obesity is associated with poorer walking capacity and adverse cardiovascular characteristics in this population [29], while obesity has also been associated with cardiovascular events among patients with PAD [30].

Fasting glucose and HbA1c did not change significantly during follow-up. This finding should not be interpreted as evidence that exercise has no metabolic effect. Exercise-related changes in glycemic control depend on exercise volume and intensity, baseline metabolic status, dietary intake, and pharmacological treatment. Previous meta-analyses have demonstrated that structured exercise can improve glycemic control in patients with type 2 diabetes, although the magnitude of benefit is related to exercise volume and intervention characteristics [31,32]. The relatively stable glycemic profile in our cohort may therefore reflect adequate baseline control, ongoing antidiabetic therapy, and the absence of a standardized nutritional intervention.

A numerical decline was observed in LDL-C concentrations, with the median value decreasing from 105.68 mg/dL at baseline to 75.05 mg/dL at 6 months. However, this change of 30.63 mg/dL did not reach statistical significance in the overall longitudinal analysis (*p* = 0.103). This numerical decline should therefore be interpreted cautiously and may have been influenced, at least in part, by concomitant lipid-lowering treatment, as statin therapy was initiated or optimized in some participants during the study period. Therefore, the observed change in LDL-C cannot be attributed specifically to the TR intervention. TC decreased significantly during follow-up. This observation is broadly consistent with recent evidence demonstrating favorable effects of exercise training on blood lipid concentrations [33]. However, the lipid changes observed in the present study cannot be attributed exclusively to walking because lipid-lowering therapy was initiated or optimized in some participants.

HDL-C remained relatively stable throughout follow-up. This is compatible with evidence suggesting that changes in HDL-C following exercise are generally modest and may depend on exercise characteristics and baseline metabolic status [34].

TG levels did not change significantly. This is compatible with the heterogeneous effects of exercise on TG concentrations reported in the literature [33].

Overall, the changes in blood pressure, BMI, and lipid profile observed in this study are clinically interesting but should be interpreted within the context of comprehensive CVR management rather than as isolated effects of TR.

### 4.4. Inflammatory and Hematological Parameters

The absence of significant changes in CRP and most traditional inflammatory markers should be interpreted in the context of the relatively stable inflammatory profile of the cohort at baseline. PAD is characterized by persistent low-grade inflammation, which contributes to atherosclerotic progression and CVR [35].

Previous evidence regarding the effect of exercise on CRP is heterogeneous. A systematic review and meta-analysis demonstrated that exercise training can reduce CRP concentrations under certain conditions [36], while other studies have reported more limited effects, particularly when baseline inflammatory activity is low [37]. Similarly, combining exercise with weight-loss interventions may produce more pronounced changes in inflammatory markers [38].

The absence of significant changes in CRP, ESR, or WBC in the present study should therefore not be interpreted as evidence of a lack of biological effect. Rather, it may indicate that the inflammatory response to a moderate-intensity home-based walking intervention was insufficient to produce measurable changes in conventional systemic biomarkers over 6 months.

Hb and serum iron remained relatively stable. This finding is consistent with previous studies suggesting that moderate exercise does not necessarily alter serum iron status or Hb concentrations in individuals without clinically significant serum iron deficiency [39,40]. Maintaining stable Hb levels is nevertheless clinically relevant in PAD because oxygen delivery to ischemic skeletal muscle contributes directly to exercise tolerance.

### 4.5. Renal Function and Carotid Atherosclerosis

Renal function remained relatively stable during follow-up, with no significant changes in Cr or Cl Cr. These findings are consistent with previous evidence suggesting that walking-based exercise does not adversely affect renal function in patients with CKD. In a retrospective study of patients with PAD and CKD, Piva et al. reported that HBET was associated with less progressive renal function deterioration over a 5-year period [41]. Similarly, a recent study of rhythmic walking in patients with CKD stages 2–3 suggested that low-intensity walking may be compatible with preservation of renal function [42].

The lack of significant changes in Cr and Cl Cr in the present study is therefore reassuring. Changes in serum urea and uric acid should nevertheless be interpreted cautiously because these parameters are influenced by hydration, dietary intake, protein metabolism, renal function, and exercise-related metabolic changes [43,44,45].

IMT ICA also remained relatively stable. IMT is associated with CVR, although its reproducibility and prognostic value may differ according to the arterial segment assessed [46]. Evidence suggests that exercise may produce modest reductions in carotid IMT, particularly with longer interventions, although the magnitude of change is generally small [47]. Therefore, the absence of a significant change in ICA IMT over 6 months is not unexpected and should not be interpreted as evidence against a vascular benefit of exercise.

### 4.6. Functional Walking Capacity

The improvement in 6MWT performance was one of the most clinically relevant secondary findings. Median 6MWT distance increased by 55 m between baseline and 6 months. This magnitude of improvement exceeds the threshold previously proposed as clinically important in patients with PAD and may therefore represent a clinically meaningful change. Recent evidence suggests that a change of approximately 20 m in 6MWT distance may represent a clinically important improvement in patients with lower-extremity PAD [48]. Therefore, the 55 m increase observed in the present study exceeds this threshold and may reflect a meaningful improvement in functional walking capacity.

The findings are consistent with previous evidence supporting home-based walking interventions in PAD. A randomized clinical trial demonstrated that a home-based walking behavior-change intervention improved walking outcomes compared with usual care [24]. More broadly, systematic review and meta-analysis evidence supports exercise training as an effective strategy for improving functional performance in PAD [49].

The progressive increase in the IC onset threshold is also consistent with improved walking tolerance. Exercise training may improve peripheral muscle function, metabolic efficiency, endothelial function, and tolerance to ischemic symptoms without necessarily producing measurable changes in the underlying arterial obstruction.

Taken together, the concurrent improvement in walking distance and IC onset threshold suggests that the intervention may have translated into greater functional walking tolerance, although the absence of a control group prevents attribution of these changes specifically to TR.

### 4.7. Ankle–Brachial Index and Cardiopulmonary Exercise Testing

ABI remained relatively stable throughout follow-up. This finding is not unexpected because ABI primarily reflects the hemodynamic severity of arterial obstruction and may not capture functional adaptations induced by exercise.

The absence of significant changes in ABI alongside improved walking performance is therefore compatible with previous exercise studies in PAD demonstrating improvements in functional capacity without parallel changes in resting vascular indices [49,50].

Although structured exercise may improve aerobic capacity in PAD, the magnitude of improvement depends on exercise intensity, duration, and baseline functional status [50]. The selected CPET parameters remained relatively stable. In particular, maximum workload and VE/VCO_2_ did not change significantly. One possible explanation for the absence of significant changes in CPET parameters is the difference between the modality of training and the modality of assessment. The intervention consisted primarily of walking exercise, whereas CPET was performed on a cycle ergometer, and exercise-induced adaptations may be partly specific to the mode of exercise. Consequently, improvements in walking-specific functional capacity may have been better captured by the 6MWT than by cycle ergometer-based CPET. In addition, as the intervention was not standardized through real-time supervision, the actual intensity of walking may have varied between participants, which could have further limited the magnitude of changes detectable during CPET.

This may indicate that the functional improvement observed in the 6MWT was not accompanied by a measurable change in the selected cardiopulmonary parameters. It may instead reflect peripheral adaptations, improved walking efficiency, improved tolerance to ischemic symptoms, or behavioral changes.

The slight, non-significant reduction in VE/VCO_2_ observed during follow-up should therefore be considered exploratory. Ventilatory efficiency may improve with exercise training, but clinically meaningful changes may require longer or more intensive exercise exposure, and interpretation should consider the underlying cardiovascular phenotype [51].

The relatively stable target HR across the three assessments is also consistent with previous findings in patients with PAD. Brenner et al. reported that low-intensity exercise training increased HR variability in patients with PAD without necessarily producing major changes in maximal HR, suggesting that exercise-related adaptation may occur through autonomic mechanisms rather than through an increase in maximal chronotropic response [52].

### 4.8. Health-Related Quality of Life Discrepancies

All three disease-specific quality of life instruments demonstrated statistically significant longitudinal variations over the 6-month protocol, although the specific tempo and magnitude of these improvements differed across scales. Both the PADQOL and QOLPAD indices captured progressive improvements from baseline through the 6-month checkpoint. In contrast, the VascuQoL-6 score demonstrated significant improvements at the 3-month assessment and between 3 and 6 months; however, the direct baseline-to-6-month comparison did not reach statistical significance (*p* = 0.068; Table 4). This discrepancy may reflect differences in questionnaire structure and sensitivity to change, as well as the reduced number of participants available for some pairwise comparisons. Four participants missed the 3-month clinical reassessment, and two missed the 6-month reassessment, although all six continued the TR intervention. These missing assessments may have reduced the statistical power of the direct baseline-to-6-month comparison. The VascuQoL-6 findings should therefore be interpreted cautiously. The absence of statistical significance in the direct baseline-to-6-month comparison does not necessarily indicate an absence of change in HRQoL. Rather, it may reflect the sensitivity of a brief, 6-item instrument to missing data within a non-parametric analytical framework. Overall, the upward trend across all three instruments is consistent with previous evidence indicating that exercise-based rehabilitation may improve psychosocial, physical, and emotional aspects of well-being in patients with vascular disease.

Our findings are consistent with previous evidence that exercise-based rehabilitation improves HRQoL in patients with PAD. A systematic review and meta-analysis demonstrated beneficial effects of exercise training on HRQoL [24]. Similarly, randomized clinical trial data suggest that both aerobic and combined aerobic-resistance training may improve walking performance and HRQoL [53].

The use of disease-specific instruments is particularly relevant because PAD affects physical functioning, symptoms, emotional well-being, and social participation. The PADQOL, for example, was specifically developed to capture the multidimensional impact of PAD on HRQoL [20].

However, statistical significance should not automatically be equated with clinical significance. The present study did not prespecify minimal clinically important differences for the exact questionnaire versions used. Therefore, the magnitude of the observed changes should be interpreted cautiously.

### 4.9. Cardiovascular Risk

The estimated 10-year CVR changed significantly across the three assessment points, with a significant difference between baseline and 6 months.

However, this result should be interpreted cautiously. CVR scores are influenced by multiple variables, including blood pressure, lipid levels, smoking status, diabetes, and other clinical characteristics. Changes in the score may therefore occur because of changes in individual risk factors rather than because of the TR intervention.

Previous rehabilitation studies in patients with PAD have demonstrated improvements in CVR factors following exercise interventions [54]. Nevertheless, the present findings reinforce the importance of considering exercise as one component of comprehensive CVR management rather than as an isolated treatment.

### 4.10. Adherence and Daily Physical Activity

Analysis of adherence revealed a divergent pattern across the different indicators used to evaluate participation in the intervention. During the first 3 months, participants achieved the step target prescribed for the following week more frequently than during the final 3 months (58.33% vs. 16.66%). Similarly, the proportion of weeks in which participants increased their activity relative to the preceding week decreased from 66.67% during the first period of the study to 50.00% during the second one.

Despite this decline in progressive target attainment, overall physical activity increased during the intervention. The proportion of weeks in which participants exceeded an average of 5000 steps/day increased from 75.00% during the first 3 months to 91.66% during the final 3 months. Moreover, median average daily step count increased from 5868 to 6905 steps/day. These findings indicate that the reduction in the proportion of successfully achieved progression targets was not accompanied by a reduction in absolute physical activity.

This apparent discrepancy is clinically relevant because the intervention used individualized and progressively increasing step targets. As participants increased their activity, the subsequent targets became progressively more demanding. Consequently, a lower proportion of achieved targets over time may reflect the increasing difficulty of maintaining continuous progression rather than declining engagement with the rehabilitation program. In this context, target attainment alone may provide an incomplete representation of adherence.

The increase in daily step count observed in the present study is consistent with previous evidence indicating that patients with PAD generally have lower levels of daily physical activity than individuals without PAD and may experience difficulties reaching higher levels of ambulatory activity [55]. Daily step volume may nevertheless represent a clinically meaningful indicator of habitual physical activity, particularly when interpreted in relation to the patient’s baseline functional capacity.

Evidence from large population-based studies also suggests a graded association between daily step counts and cardiovascular health outcomes, with clinically relevant benefits occurring below the traditionally promoted threshold of 10,000 steps/day [56]. Similar associations have been reported for all-cause mortality, cardiovascular disease, and metabolic outcomes [57]. These findings support the use of individualized activity targets rather than a universal 10,000-step goal, particularly in patients with PAD whose walking capacity may be limited by IC.

Taken together, the present findings suggest that adherence should not be interpreted solely according to whether participants achieved their progressively increasing weekly targets. A more comprehensive assessment should incorporate absolute activity volume, clinically relevant step thresholds, and achievement of individualized progression targets. The observed increase in daily step count, despite reduced attainment of progressively demanding targets, suggests that participants remained engaged with the intervention, while absolute habitual physical activity increased over the 6-month period.

### 4.11. Clinical Context

The findings are relevant to current PAD management because international guidelines emphasize structured exercise as a core component of care. The 2024 ESC Guidelines recognize both supervised and structured community-based exercise programs, including home-based programs, as approaches to improve functional status, walking performance, and HRQoL [5].

The present study therefore does not propose TR as a replacement for SET in all patients. Instead, it explores whether a digitally supported home-based model can be implemented in patients who may have limited access to conventional rehabilitation services.

The absence of real-time supervision is also relevant. The intervention evaluated in this study represents a low-resource model based on wearable monitoring and telephone counseling. Its feasibility may therefore be particularly relevant in settings where specialized SETs are not widely available.

### 4.12. Strengths and Limitations

This study has several strengths.

First, it prospectively evaluated a pragmatic home-based rehabilitation model over 6 months.

Second, physical activity was monitored using objective wearable-device data rather than relying exclusively on self-reported exercise.

Third, weekly telephone counseling provided a structured behavioral component.

Fourth, the study included functional, cardiopulmonary, vascular, laboratory, and disease-specific quality of life assessments.

Several limitations should nevertheless be considered.

First, the absence of a control group substantially limits causal inference. Changes observed during follow-up may have resulted from natural disease variation, regression to the mean, medication changes, dietary counseling, increased attention from healthcare professionals, or other unmeasured factors.

Second, participants were recruited as a convenience sample from a cardiovascular rehabilitation setting. Individuals who volunteered for a 6-month program requiring smartwatch use and weekly telephone contact may have been more motivated, technologically capable, or functionally healthier than the broader PAD population. Selection bias may therefore have contributed to the observed adherence.

Third, no primary efficacy endpoint had been prespecified. The study was designed primarily to assess feasibility and adherence, while the clinical outcomes were exploratory. The large number of variables analyzed increases the possibility of type I error despite the use of Bonferroni correction for pairwise comparisons.

Fourth, missing clinical outcome data occurred at follow-up. Four participants did not attend the 3-month clinical reassessment, and two did not attend the 6-month reassessment, although all six participants continued the TR intervention. The missing clinical measurements therefore reflect missed reassessment visits rather than discontinuation of the intervention. These missing data should nevertheless be reported transparently and handled consistently across analyses.

Fifth, the Xiaomi Smart Band 8 was not used as a research-grade activity-monitoring device. Wrist-worn step counters may be affected by arm movement, slow gait, device positioning, and other factors. Therefore, recorded steps should be interpreted as estimates of overall physical activity rather than exact measurements of exercise volume.

Sixth, the intervention was not standardized through real-time supervision. The actual intensity of walking may therefore have varied between participants.

Seventh, dietary intake and medication adjustments were not controlled experimentally. Consequently, changes in blood pressure, BMI, lipid profile, and other metabolic variables cannot be attributed solely to the rehabilitation program.

Eighth, the follow-up period was limited to 6 months, preventing conclusions regarding long-term adherence and sustainability.

Finally, the results may not be generalizable to patients with chronic limb-threatening ischemia, severe cognitive impairment, major locomotor limitations, or patients without access to smartphones or wearable devices.

### 4.13. Clinical Implications

The principal clinical implication of this study is not that TR has been proven to improve cardiovascular or functional outcomes. Rather, the findings indicate that a structured home-based rehabilitation model incorporating wearable activity monitoring and regular telephone counseling can be implemented over a 6-month period in selected patients with PAD.

Home-based TR combining wearable activity monitoring and structured telephone counseling may therefore represent a practical rehabilitation strategy for selected patients with limited access to supervised exercise programs.

This approach may be particularly useful in healthcare systems where access to SET is limited.

The observed increase in overall daily activity, together with the improvement in 6MWT performance and HRQoL, provides a rationale for further investigation. However, implementation should include appropriate patient selection, medical assessment, individualized exercise prescription, monitoring of symptoms, and mechanisms for escalation to supervised care when necessary.

### 4.14. Future Research

Future studies should use randomized controlled designs comparing digitally supported home-based rehabilitation with usual care, SET, or alternative HBET. Larger studies should prespecify primary clinical or feasibility endpoints and standardized criteria for adherence, retention, intervention completion, and device use. Longer follow-up and validated measures of exercise intensity, perceived exertion, claudication severity, and adherence are needed to determine whether improvements in walking performance and health-related quality of life (HRQoL) are sustained and clinically meaningful. Further research should also validate wearable activity monitors in patients with peripheral arterial disease (PAD) and evaluate the cost-effectiveness and scalability of digitally supported home-based rehabilitation.

## 5. Conclusions

This prospective, single-arm study indicates that a 6-month home-based TR model combining progressive walking, wearable activity monitoring, and weekly telephone counseling was feasible in patients with PAD.

Absolute physical activity increased over follow-up, although achievement of progressively increasing weekly step targets declined.

Secondary exploratory analyses showed significant improvements in IC onset threshold, 6MWT distance, and disease-specific HRQoL, while ABI and selected CPET parameters remained stable. The median 6MWT distance increased by 55 m from baseline to 6 months (*p* = 0.001).

Given the uncontrolled single-arm design, these longitudinal changes cannot be attributed specifically to the TR intervention. Larger randomized controlled studies are needed to determine the effectiveness and long-term sustainability of this rehabilitation model.

## Figures and Tables

**Figure 1:**
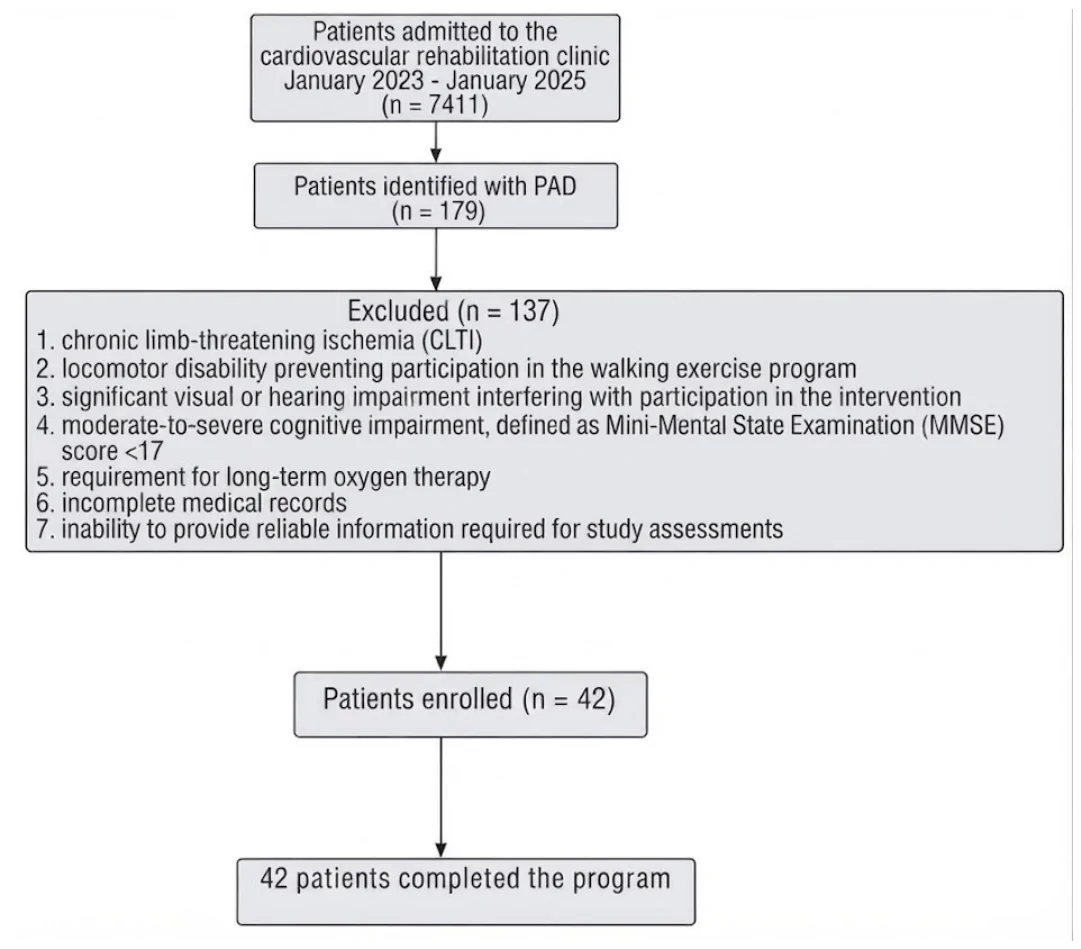
Patient enrollment.

**Figure 2:**
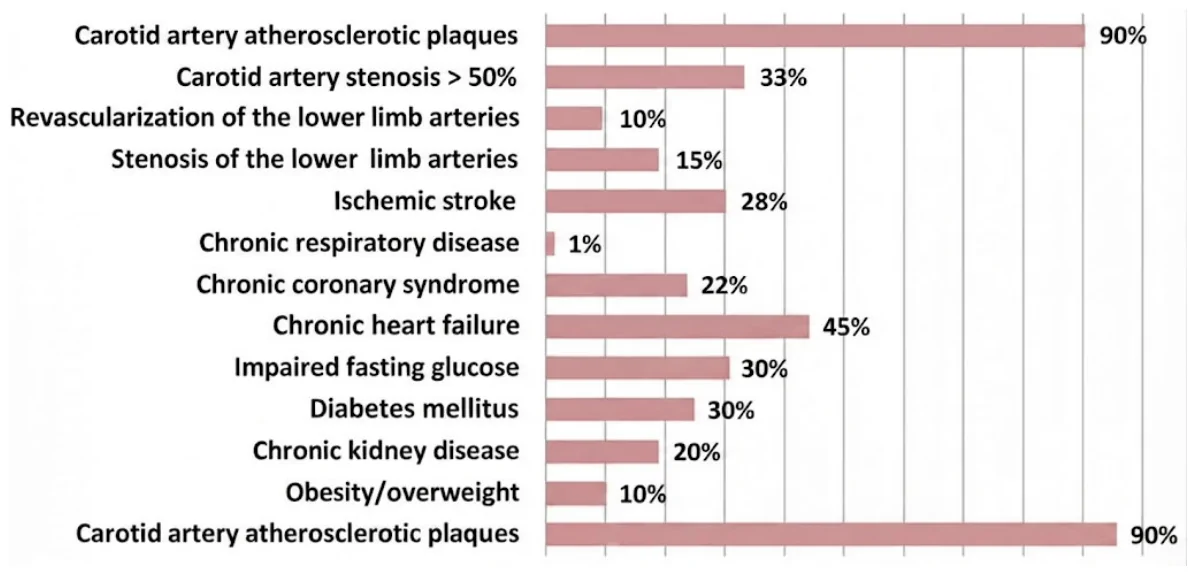
Comorbidities.

**Table 1:** General characteristics of patients.

Variable	*n* (%)
Demographics	
Male	31 (73.8%)
Female	11 (26.2%)
Years Since PAD Diagnosis	
<1 year	17 (40.4%)
1–5 years	11 (26.2%)
>5 years	14 (33.4%)
PAD Classification	
Asymptomatic PAD	5 (11.9%)
Symptomatic exertional PAD	37 (88.1%)
Lifestyle Factors	
Current smoker	11 (26.2%)
Former smoker	23 (54.7%)
Chronic alcohol consumption	9 (21.4%)
Occasional alcohol consumption	12 (28.5%)

Abbreviations: PAD—peripheral arterial disease.

**Table 2:** Clinical and paraclinical assessments at baseline, 3 months, and 6 months.

Parameter	Initial Assessment Median [IQR]	3 Months Median [IQR]	6 Months Median [IQR]	Friedman Test (Chi-Square, *p*)	Post hoc Pairwise Comparisons (Wilcoxon Test) Pair: Z, *p*-Value, [95% CI of Diff], Effect Size [r]
SBP (mmHg)	140.00 [131.00–150.00]	128.00 [114.50–146.00]	131.50 [122.00–140.00]	Chi-square = 10.126, *p* = 0.006	Init vs. 3 M: Z = −2.203, *p* = 0.028, CI: [−13.00 to −0.50], r = 0.344 3 M vs. 6 M: Z = −0.244, *p* = 0.808, CI: [−2.50 to −0.50], r = 0.039 Init vs. 6 M: Z = −3.351, *p* = 0.001, CI: [−14.50 to −5.00], r = 0.530
DBP (mmHg)	80.00 [72.00–86.00]	80.00 [71.50–83.50]	76.50 [72.00–81.50]	Chi-square = 2.973, *p* = 0.226	Wilcoxon test was not conducted
BMI (kg/m^2^)	29.75 [22.95–32.04]	28.84 [26.08–31.80]	28.65 [25.37–31.97]	Chi-square = 22.817, *p* ˂ 0.001	Init vs. 3 M: Z = −2.156, *p* = 0.031, CI: [−0.63 to 0.00], r = 0.337 3 M vs. 6 M: Z = −3.000, *p* = 0.003, CI: [−0.80 to −0.05], r = 0.469 Init vs. 6 M: Z = −4.280, *p* ˂ 0.001, CI: [−1.24 to −0.48], r = 0.668
Fasting glucose (mg/dL)	116.30 [106.00–139.60]	115.41 [100.00–133.36]	110.80 [100.92–136.67]	Chi-square = 1.400, *p* = 0.497	Wilcoxon test was not conducted
HbA1c (%)	6.42 [5.89–7.61]	6.34 [5.88–7.45]	6.54 [5.77–7.53]	Chi-square = 1.368, *p* = 0.504	Wilcoxon test was not conducted
TC (mg/dL)	180.00 [141.00–217.00]	147.00 [133.00–181.50]	147.00 [117.25–181.75]	Chi-square = 6.650, *p* = 0.036	Init vs. 3 M: Z = −2.177, *p* = 0.029, CI: [−41.00 to −3.00], r = 0.340 3 M vs. 6 M: Z = −1.163, *p* = 0.245, CI: [−18.00 to 6.00], r = 0.179 Init vs. 6 M: Z = −3.176, *p* = 0.001, CI: [−48.00 to −10.00], r = 0.490
HDL-C (mg/dL)	55.20 [48.10–64.00]	50.73 [44.85–68.00]	50.95 [42.17–60.67]	Chi-square = 4.200, *p* = 0.122	Wilcoxon test was not conducted
LDL-C (mg/dL)	105.68 [81.50–135.00]	86.40 [65.30–114.60]	75.05 [57.00–106.42]	Chi-square = 4.550, *p* = 0.103	Wilcoxon test was not conducted
TG (mg/dL)	122.90 [98.90–162.80]	107.60 [89.45–153.25]	113.79 [86.25–146.47]	Chi-square = 3.950, *p* = 0.139	Wilcoxon test was not conducted
CRP (mg/dL)	0.48 [0.23–0.97]	0.58 [0.22–0.91]	0.39 [0.20–1.02]	Chi-square = 0.050, *p* = 0.975	Wilcoxon test was not conducted
ESR (mm/h)	12.00 [9.00–21.00]	12.00 [8.25–22.00]	12.50 [7.00–25.00]	Chi-square = 0.416, *p* = 0.812	Wilcoxon test was not conducted
WBC (number/mm^3^)	7270.00 [6000.00–9100.00]	7300.00 [6055.00–8890.00]	7295.00 [6050.00–8605.00]	Chi-square = 0.264, *p* = 0.876	Wilcoxon test was not conducted
Serum iron (µg/dL)	80.10 [51.00–95.60]	71.71 [59.27–89.55]	72.70 [60.87–91.32]	Chi-square = 2.667, *p* = 0.264	Wilcoxon test was not conducted
Hb (g/dL)	13.90 [12.90–14.70]	14.00 [13.00–14.90]	13.85 [12.70–14.75]	Chi-square = 2.823, *p* = 0.244	Wilcoxon test was not conducted
Cl Cr (mL/min)	66.18 [53.89–74.16]	66.26 [57.83–72.28]	64.65 [51.46–71.98]	Chi-square = 0.200, *p* = 0.905	Wilcoxon test was not conducted
Cr (mg/dL)	1.12 [0.98–1.23]	1.07 [0.96–1.24]	1.10 [0.98–1.32]	Chi-square = 0.052, *p* = 0.975	Wilcoxon test was not conducted
Urea (mg/dL)	42.60 [35.70–51.90]	37.58 [31.47–47.70]	39.65 [31.05–49.50]	Chi-square = 4.051, *p* = 0.132	Wilcoxon test was not conducted
Uric acid (mg/dL)	5.61 [4.29–6.31]	5.57 [4.39–6.12]	5.34 [4.31–6.32]	Chi-square = 1.296, *p* = 0.523	Wilcoxon test was not conducted
Right IMT ICA	0.80 [0.70–1.00]	0.80 [0.70–0.90]	0.90 [0.72–1.07]	Chi-square = 1.436, *p* = 0.488	Wilcoxon test was not conducted
Left IMT ICA	0.90 [0.70–1.00]	0.85 [0.70–0.90]	0.90 [0.70–1.00]	Chi-square = 2.000, *p* = 0.368	Wilcoxon test was not conducted

Abbreviations: IC—intermittent claudication, ABI—ankle–brachial index, BMI—body mass index, Cr—Creatinine, Cl Cr—Creatinine clearance, CRP—C-reactive protein, DBP—diastolic blood pressure, ESR—erythrocyte sedimentation rate, Hb—hemoglobin, HbA1c—glycated hemoglobin, HDL-C—high-density lipoproteins, ICA—internal carotid artery, IMT—intima-media thickness, LDL-C—low-density lipoproteins, SBP—systolic blood pressure, TC—total cholesterol, TG—triglycerides, WBC—white blood cells.

**Table 3:** Assessment of functional status, cardiovascular risk, and prognosis.

Parameter	Initial Assessment Median [IQR]	3 Months Median [IQR]	6 Months Median [IQR]	Friedman Test (χ^2^, *p*-Value)	Post hoc Pairwise Comparisons (Wilcoxon Test) Pair: Z, *p*-Value, 95% CI of Diff, Effect Size [r]
IC onset threshold (m)	200.00 [100.00–400.00]	300.00 [200.00–650.00]	500.00 [200.00–1000.00]	χ^2^ = 55.866, *p* ˂ 0.001	Init vs. 3 M: Z = −4.798, *p* ˂ 0.001, CI: [50.00–170.00], r = 0.749 3 M vs. 6 M: Z = −3.779, *p* ˂ 0.001, CI: [50.00–170.00], r = 0.590 Init vs. 6 M: Z = −5.166, *p* ˂ 0.001, CI: [120.00–250.00], r = 0.797
Right ABI	0.76 [0.60–0.92]	0.75 [0.62–0.95]	0.77 [0.64–0.95]	χ^2^ = 1.382, *p* = 0.501	Wilcoxon test was not conducted
Left ABI	0.71 [0.63–0.86]	0.74 [0.63–0.94]	0.76 [0.61–0.91]	χ^2^ = 5.503, *p* = 0.064	Wilcoxon test was not conducted
Distance (m)	390.00 [300.00–480.00]	420.00 [360.00–480.00]	445.00 [377.50–493.75]	χ^2^ = 14.746, *p* = 0.001	Init vs. 3 M: Z = −1.567, *p* = 0.117, CI: [−5.00–37.50], r = 0.245 3 M vs. 6 M: Z = −3.658, *p* ˂ 0.001, CI: [12.50–40.00], r = 0.578 Init vs. 6 M: Z = −3.375, *p* = 0.001, CI: [17.50–65.00], r = 0.534
Percentage achieved (%)	61.80 [47.20–72.30]	62.50 [55.40–75.10]	69.05 [56.95–77.30]	χ^2^ = 17.568, *p* ˂ 0.001	Init vs. 3 M: Z = −1.382, *p* = 0.167, CI: [−0.92–5.70], r = 0.216 3 M vs. 6 M: Z = −3.661, *p* ˂ 0.001, CI: [2.15–6.36], r = 0.579 Init vs. 6 M: Z = −3.448, *p* = 0.001, CI: [2.65–10.60], r = 0.545
Level of effort (Watt)	78.00 [66.00–114.25]	79.00 [70.00–111.25]	82.00 [78.00–95.00]	χ^2^ = 0.950, *p* = 0.622	Wilcoxon test was not conducted
VE/VCO_2_	42.15 [30.92–48.33]	40.53 [34.33–45.83]	36.90 [30.30–42.09]	χ^2^ = 0.549, *p* = 0.460	Init vs. 6 M: Z = −0.600, *p* = 0.549, CI: [−8.54–4.50], r = 0.113 (Other pairs not conducted)
Target HR (bpm)	90.00 [78.50–96.75]	94.00 [85.00–102.00]	93.00 [85.00–100.00]	χ^2^ = 3.607, *p* = 0.165	Wilcoxon test was not conducted
CVR at 10 years (%)	22.30 [12.40–35.50]	18.50 [8.55–34.80]	22.80 [11.20–33.70]	χ^2^ = 7.840, *p* = 0.020	Init vs. 3 M: Z = −1.950, *p* = 0.051, CI: [−3.90–0.00], r = 0.305 3 M vs. 6 M: Z = −0.921, *p* = 0.357, CI: [−1.10–2.90], r = 0.146 Init vs. 6 M: Z = −2.313, *p* = 0.021, CI: [−3.05 to −0.35], r = 0.366

Abbreviations: ABI—ankle–brachial index, CVR—cardiovascular risk, HR—heart rate, IC—intermittent claudication, VE/VCO_2_—minute ventilation–carbon dioxide production ratio.

**Table 4:** Assessment of health-related quality of life.

Parameter	Initial Assessment Median [IQR]	3 Months Median [IQR]	6 Months Median [IQR]	Friedman Test (χ^2^, *p*-Value)	Post hoc Pairwise Comparisons (Wilcoxon Test) Pair: Z, *p*-Value, 95% CI of Diff, Effect Size [r]
PADQOL (points)	125.00 [98.00–142.00]	143.00 [120.50–162.00]	154.50 [135.00–175.00]	χ^2^ = 33.365, *p* ˂ 0.001	Init vs. 3 M: Z = −4.215, *p* ˂ 0.001, CI: [12.00–28.00], r = 0.658 3 M vs. 6 M: Z = −2.302, *p* = 0.021, CI: [0.50–3.50], r = 0.364 Init vs. 6 M: Z = −4.640, *p* ˂ 0.001, CI: [20.00–40.50], r = 0.721
QOLPAD (points)	40.00 [34.00–45.00]	45.00 [41.00–48.00]	46.50 [43.00–50.50]	χ^2^ = 19.557, *p* ˂ 0.001	Init vs. 3 M: Z = −3.597, *p* ˂ 0.001, CI: [2.50–7.00], r = 0.562 3 M vs. 6 M: Z = −2.138, *p* = 0.033, CI: [0.50–3.50], r = 0.338 Init vs. 6 M: Z = −4.356, *p* ˂ 0.001, CI: [4.00–8.50], r = 0.689
VascuQoL-6 (points)	14.00 [11.00–17.00]	18.00 [15.00–20.00]	18.50 [16.00–22.00]	χ^2^ = 31.483, *p* ˂ 0.001	Init vs. 3 M: Z = −4.401, *p* ˂ 0.001, CI: [4.50–5.50], r = 0.687 3 M vs. 6 M: Z = −4.562, *p* ˂ 0.001, CI: [3.00–6.00], r = 0.721 Init vs. 6 M: Z = −1.822, *p* = 0.068, CI: [0.00–2.00], r = 0.288

Abbreviations: PADQOL—Peripheral artery disease quality of life questionnaire; QOLPAD—Quality of life of patients with peripheral artery disease questionnaire; VascuQoL-6—Vascular quality of life questionnaire.

**Table 5:** Adherence to the telerehabilitation program.

Parameter	Comparison	Median 1 [IQR]	Median 2 [IQR]	*Z*	*p*-Value	95% CI of Diff	Effect Size [*r*]
Reaching the average daily step target set for the following week (% of the time)	First 3 months vs. last 3 months	58.33 [33.30–83.33]	16.66 [0.00–33.33]	−5.337	˂0.001	[−41.68–−25.00]	0.814
Increase in average daily steps compared to the previous week (% of the time)	First 3 months vs. last 3 months	66.67 [58.33–66.67]	50.00 [50.00–58.33]	−3.600	˂0.001	[−12.50–−4.17]	0.549
Average steps over 5000 (steps/day)	First 3 months vs. last 3 months	75.00 [25.00–100.00]	91.66 [50.00–100.00]	−1.975	0.048	[−12.50–−4.17]	0.301
Average steps over 10,000 (steps/day)	First 3 months vs. last 3 months	0.00 [0.00–0.00]	0.00 [0.00–41.66]	−2.719	0.007	[00.00–12.51]	0.415
Average steps over 12 weeks (steps/day)	First 3 months vs. last 3 months	5868.00 [4469.00–7620.00]	6905.00 [5368.00–8929.00]	−3.647	˂0.001	[531.50–1383.00]	0.556

## Data Availability

The date presented presented in this study are available on request from the corresponding author due to privacy and ethical restrictions.

## Abbreviations

The following abbreviations are used in this manuscript:

6MWT	6 min walk test
ABI	Ankle-brachial index
ATP	Adenosine triphosphate
ATS	Atherosclerosis
BMI	Body mass index
CCS	Chronic coronary syndrome
CKD	Chronic kidney disease
Cl Cr	Creatinine clearance
CPET	Cardiopulmonary exercise testing
Cr	Serum creatinine
CRP	C-reactive protein
CT	Computed tomography
CVR	Cardiovascular risk (CVR)
DBP	Diastolic blood pressure
ESR	Erythrocyte sedimentation rate
Hb	Hemoglobin
HbA1c	Glycated hemoglobin
HBET	Home-based exercise therapy
HDL-C	High-density lipoproteins
HF	Heart failure
HONOR	Home-Based Observed/Non-supervised Networked Objective Randomized trial
HR	Heart rate
IC	Intermittent claudication
IL-6	Interleukin 6
IMT ICA	Intima-media thickness of the internal carotid arteries
IQR	Interquartile range
LCAT	Lecithin-cholesterol acyltransferase
LDL-C	Low-density lipoproteins
LITE	Low-Intensity vs. High-Intensity Home-Based Walking Exercise trial
MACE	Major adverse cardiovascular events
MiRNA-126	MicroRNA-126
NAD^+^	Oxidized adenine dinucleotide
NADH	Reduced nicotinamide adenine dinucleotide
PAD	Peripheral artery disease of the lower limbs
PADQOL	Peripheral Artery Disease Quality of Life
PCr	Phosphocreatine
PET	Positron emission tomography
HRQoL	Health-related quality of life
QOLPAD	Quality of Life of Patients with Peripheral Artery Disease
SBP	Systolic blood pressure
SET	Supervised exercise therapy
SMART	Second Manifestations of ARTerial disease study
TC	Total cholesterol
TEXTPAD	Telehealth Exercise Training in Peripheral Arterial Disease study
TG	Triglyceride
TNF	Tumor necrosis factor α
TR	Telerehabilitation
VascuQoL-6	Vascular Quality of Life 6
VE/VCO2	Minute ventilation–carbon dioxide production ratio
VEGF	Vascular endothelial growth factor
WBC	White blood cell
